# Assessment of Intermingled Phlegm and Blood Stasis Syndrome in Coronary Heart Disease: Development of a Diagnostic Scale

**DOI:** 10.1155/2018/4683431

**Published:** 2018-10-24

**Authors:** Xuan Zhou, Xian-tao Li, Xiao-qi Liu, Bing Wang, Ge Fang

**Affiliations:** School of Basic Medical Science, Guangzhou University of Chinese Medicine, Guangzhou 510006, China

## Abstract

**Background:**

Intermingled Phlegm and Blood Stasis Syndrome (IPBSS) is a common feature in patients with coronary heart disease (CHD). In clinical practice, the diagnostic agreement of clinical doctor of Chinese Medicine (CM) is poor. We previously developed a IPBSS diagnostic scale for use by general practitioner.

**Objectives:**

To assess a IPBSS diagnostic scale that we previously developed for use by non-experts.

**Methods:**

This is a multicenter, prospective study involving eight study sites across China. Eligible patients were adults (≥18 years) with CHD as demonstrated by a history of myocardial infarction, stenosis, or past coronary revascularization. IPBSS was assessed using a scale that consisted of 14 items in two domains (e.g., phlegm and blood stasis). The score range for each item was 0 to 3 points. Maximum total score was 72 points. Diagnostic accuracy was verified using consensus opinion by two independent experts as reference.

**Results:**

A total of 1,142 CHD patients were included. IPBSS was established in 729 subjects using the IPBSS diagnostic scale. In ROC curve analyses, at the optimal cut-off of 25.5, the sensitivity and specificity of the IPBSS scale were 67.6% and 72.4%, respectively. The area under the ROC curve was 0.741 (95%CI: 0.711-0.772).

**Conclusions:**

The newly developed IPBSS scoring system showed moderate performance in diagnosing IPBSS in CHD patients. Data from further large-scale diagnostic test accuracy studies are warranted. This trial is registered with ChiCTR-OOC-15006599.

## 1. Introduction

Coronary heart disease (CHD) is one of the major causes of mortality and morbidity in developed countries [1]. Although CHD-related mortality has gradually declined over the last decades, it remains a leading cause of death in Europe [2]. In China, the number of people diagnosed with CHD has reached a total of 11 million in 2017 [3]. Many CHD patients have been successfully managed by CM, which emphasizes pattern identification [4]. Such an approach helps to identify the underlying pathological bases of the disease and is critical in selection of optimal treatment [5]. Effective integration of pattern identification from CM with differential diagnosis from Western medicine provides a solid basis to advancing the development of a model for individualized medicine [6].

The core value of pattern identification is CM syndrome, which is identified by experts through comprehensive analysis using four diagnostic methods that are unique to the practice of CM [7]. CM syndrome may evolve during disease progression. As a result, lack of simple and standardized assessment protocol represent a major obstacle in using pattern identification in daily medical practice. Efforts have been devoted to resolve this issue, including the use of hyperspectral medical tongue images for tongue diagnosis [8], tactile sensor system to detect pulse signals for pulse diagnosis [9], and scales and questionnaires developed to standardize disease diagnosis [10]. Scales and questionnaires could be divided into four types: “different disease with same syndrome” diagnostic scale of CM syndrome [11], “disease combined syndrome (multiple)” diagnostic scale of CM syndrome [12], Syndrome Factors Diagnostic scale [13], and “disease combined syndrome (single)” diagnostic scale of CM syndrome [14]. However, these diagnostic scales are typically developed without rigorous scientific grounds in item selection and often lack validation [15]. We have previously developed a diagnostic scale to assess IPBSS in CHD [16]. In the current study, we examined the performance of this scale in a group of CHD patients.

## 2. Methods

### 2.1. Study Design

This is a multicenter, prospective diagnostic study involving eight study sites across China: Guangdong Provincial Hospital of Traditional Chinese Medicine, Hunan University of Traditional Chinese Medicine, Hubei Provincial Hospital of Traditional Chinese Medicine, Affiliated Hospital of Shandong University of Traditional Chinese Medicine, Guizhou Provincial Hospital of Traditional Chinese Medicine, Second Affiliated Hospital of Wenzhou Medical University, Changzhou City Hospital of Traditional Chinese Medicine, Tianjin University of Traditional Chinese Medicine. Enrolment commenced in October 2016 and ended in March 2018. Reporting of the results conforms to the Standards for Reporting of Diagnostic Accuracy (STARD) statement [17]. All study subjects provided written informed consent.

### 2.2. Participants

#### 2.2.1. Eligibility Criteria

Adult subjects (≥18 years) suspected of having CHD based on the presence of at least one of the followings conditions were eligible for inclusion:

1. history of myocardial infarction (ST-elevation myocardial infarction (STEMI) or non-ST-elevation myocardial infarction (NSTEMI));
2. >50% stenosis in at least one main branch of coronary arteries, as established by coronary angiography or computed tomography angiography;
3. history of percutaneous coronary intervention (PCI) or coronary artery bypass grafting (CABG) [18, 19].

Subjects with unstable angina were excluded.

### 2.3. IPBSS Scale

The diagnostic scale for IPBSS was developed based on systematic literature review [20] of best available evidence via an expert consensus-based Delphi [21] and analytic hierarchy process (AHP) [22] approach. The scoring system included a total of 14 items [16] that could be classified domains into two domains (e.g., phlegm and blood stasis). Possible range of score was 0 to 3 points for each item and 0 to 72 points for the overall assessment.

After assessment using the IPBSS diagnostic scale, symptom differentiation was conducted independently by two CM experts with the following qualifications:

- specialization in cardiovascular diseases;
- specialization in traditional Chinese medicine or integrative Chinese and Western medicine;
- having a senior professional title;
- engagement in daily clinical practice for at least 20 years.

Diagnosis of IPBSS was confirmed only on the basis where both experts reached agreement on a particular diagnostic case. If the two experts disagreed on patient status, IPBSS was considered not to be present. The performance of the IPBSS scoring system was investigated based on the consensus of opinion from the two experts. The two experts were not involved in assessing IPBSS using the scale.

### 2.4. Statistical Analysis

Statistical analysis was performed using the SPSS statistical software (IBM Corp. Released 2015. IBM SPSS Statistics for Windows, Version 23.0. Armonk, NY: IBM Corp.). Using the experts' diagnoses as the standard, we determined the receiver operating characteristic (ROC) curves for the IPBSS scores, the maximum Youden Index value, and the corresponding cut-off points [23]. In addition, the sensitivity, specificity, positive, and negative predictive values (PPV and NPV, respectively) were calculated using the following formulas:

1. Sensitivity (true positive rate, TPR) = [Patients diagnosed with IPBSS by the diagnostic scale / (Patients diagnosed with IPBSS + Patients with IPBSS but not diagnosed with IPBSS)] × 100%.
2. Specificity (true negative rate, TNR) = [Patients diagnosed with IPBSS by the diagnostic scale / (Patients with IPBSS but not diagnosed with IPBSS + Patients without IPBSS)] × 100%.
3. PPV = [Patients diagnosed with IPBSS / Patients who were tested for IPBSS] × 100%.
4. NPV = [Patients determined not to have IPBSS / Patients who were tested for IPBSS] × 100%.

Sample size was calculated as described previously [16].

## 3. Results

### 3.1. Patient Baseline Characteristics

**Figure 1:**
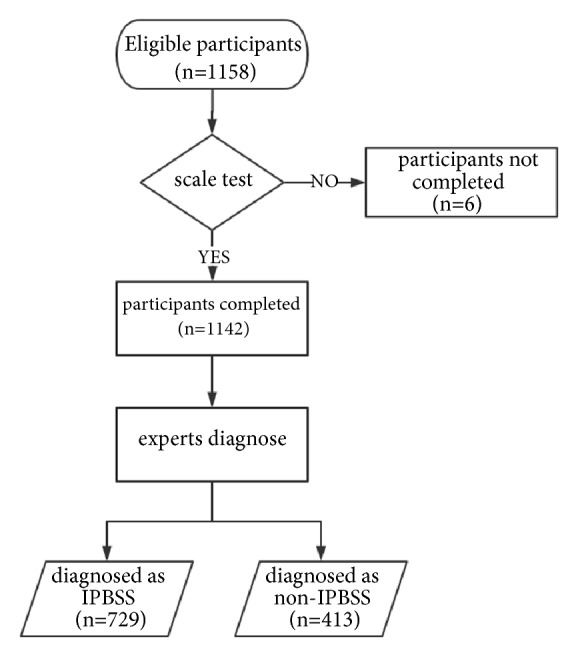
A flow diagram of the participants.

Between 1 October 2016 and 30 March 2018, a total of 1,158 eligible CHD patients were invited to participate. A total of 16 participants did not complete the IPBSS scoring. The final analysis included 1,142 subjects, with a median age of 68 years (range: 32-94 years), of whom over half were males (Figure 1)

Of the 1,142 subjects, 799 (70.0%) had hypertension (I10, ICD-10), 346 (30.3%) had type 2 diabetes mellitus (T2DM, E11)), 173 (15.1%) had hyperlipidemia (E78), 132 (11.6%) had cerebral infarction (I63), 106 (9.23%) had gastritis (K29), 90 (7.9%) had arrhythmias(I49), 72 (6.3%) had lung infection (J18), 56 (4.9%) had chronic obstructive pulmonary disease (COPD, J44), 47 (4.1%) had hyperuricemia (E79), and 47 (4.1%) had atherosclerosis (I70) (Table 1).

### 3.2. Performance of the IPBSS Scale

**Figure 2:**
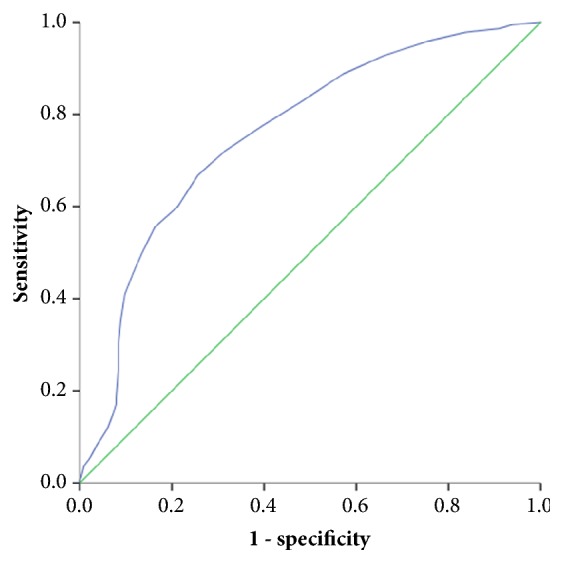
ROC analysis of the IPBSS diagnostic score (cut-off: 25.5) in assessing the presence versus absence of IPBSS. The area under the curve (AUC) was 0.741.

The total score of IPBSS scale did not conform with normal distribution (median score: 21, range 12-33). Using the ROC analysis, the optimal cut-off score of the IPBSS diagnostic scale was determined to be 25.5 (Table 2). At this cut-off, the sensitivity, specificity, PPV, and NPV of the diagnostic scale were 67.6%, 72.4%, 81.2%, and 55.9%, respectively. The area under the curve (AUC) was 0.741 (95% confidence interval (CI), 0.711-0.772) (Figure 2).

## 4. Discussion

The present study showed that, using a cut-off score of 25.5, our novel IPBSS scoring system produced reasonable sensitivity and specificity in differentiating patients with IPBSS from those without, using expert diagnoses as the reference.

Currently, only a few CM diagnostic scales are available [24], and some of these scales do not provide clear explanation or description of the methodology of their development processes [12], and some without adequate quality control in scale design procedure, data entry [25]. Therefore, utilization of these diagnostic scales poses substantial issues in terms of consistency and accuracy.

Consequently, we conducted the current study to illustrate an effective validation method of a diagnostic scale by systematically reviewing the current literature together with a AHP-Delphi consensus method to establish the list of items to be included in the IPBSS diagnostic tool, eventually reaching a total of 14 items. The items we examined were extracted from modern standards and guidelines [26, 27], which were the result of wisdom by traditional Chinese medicine researchers. And the content validity of the scale depends on the quality of these standards and guidelines. Weighting of each item was achieved via the AHP. A four-tiered classification was designed to score each item (0 to 3 points), thereby providing a total score ranging from 0 to 72 points. We subsequently developed ROC curves and observed that a cut-off score of 25.5 was associated with optimal sensitivity, specificity, PPV, and NPV.

The current study has several limitations. First, we only assessed China-based participants and did not include non-CHD subjects [28]. Our study population is, therefore, not representative and whether our IPBSS scoring system could be used to assess subjects with other conditions of other ethnicities requires further investigation. Second, we prespecified opinion from two experts as the gold standard. A true “gold standard” is hard to attain in studies exploring diagnostic test accuracy of traditional Chinese medicine. Wang and Zhou proposed the use of random effects models to estimate the diagnostic performance without a gold standard by accounting for the correlation structure among different tests or practitioners [29]. However, the models proposed are in need of further investigation to fully validate their application. We opted to use the expert consensus method not only because this method is already widely used but also because it is highly unlikely for two experts to provide entirely different opinions in CM diagnoses, given that the experts we enrolled to participate were all strong candidates with solid theoretical knowledge and practical experience. Third, our diagnostic scale is yet to be verified using a large-scale, external study cohort. For future development, we anticipate to further modify the diagnostic scale with potentially less for ease of use.

In conclusion, our novel IPBSS scoring system produced moderate performance in identifying the presence of IPBSS in Chinese CHD patients. And we will improve the scoring system in future.

## Figures and Tables

**Table 1:** Demographics of the study participants.

	IPBSS	Non-IPBSS	Overall population
	(n = 729)	(n = 413)	(n = 1142)
Gender			
Male	460 (63.1)	240 (58.1)	700 (61.3)
Age			
≤40	8 (1.1)	2 (0.5)	10 (0.9)
≤60	176 (24.1)	85 (20.6)	261 (22.9)
≤80	472 (64.8)	263 (63.6)	735 (64.4)
>80	73 (10)	63 (15.3)	136 (11.9)
Co-morbidities			
Hypertension	518 (71.1)	281 (68.03)	799 (70.0)
T2DM	207 (28.4)	139 (33.7)	346 (30.3)
Hyperlipidemia	113 (15.5)	60 (14.5)	173 (15.1)
Cerebral infarction	70 (9.6)	62 (15.0)	132 (11.6)
Gastritis	60 (8.2)	46 (11.1)	106 (9.23)
Arrhythmias	48 (6.6)	42 (10.2)	90 (7.9)
Lung infection	39 (5.3)	33 (8.00)	72 (6.3)
COPD	35 (4.8)	21 (5.08)	56 (4.9)
Hyperuricemia	29 (4.0)	18 (4.36)	47 (4.1)
Atherosclerosis	26 (3.6)	21 (5.08)	47 (4.1)

T2DM, type 2 diabetes mellitus; COPD, chronic obstructive pulmonary disease; IPBSS, Intermingled Phlegm and Blood Stasis Syndrome.

All comorbidities were diagnosed according to the International Statistical Classification of Diseases and Related Health Problems 10th Revision (ICD-10).

**Table 2:** Performance of the IPBSS scoring system at various cut-off values.

**IPBSS score**	**Sensitivity**	**Specificity**	**Youden index**
16.5	0.872	0.443	0.315
17.5	0.844	0.479	0.323
18.5	0.826	0.508	0.334
19.5	0.802	0.554	0.356
20.5	0.774	0.581	0.355
21.5	0.745	0.608	0.353
22.5	0.728	0.644	0.372
23.5	0.713	0.678	0.391
24.5	0.69	0.702	0.392
**25.5**	**0.676**	**0.724**	**0.400**
26.5	0.646	0.741	0.387
27.5	0.62	0.763	0.383
28.5	0.583	0.794	0.377
29.5	0.561	0.801	0.362
30.5	0.523	0.809	0.332
31.5	0.49	0.826	0.316

## Data Availability

The data used to support the findings of this study are available from the corresponding author upon request.

## Abbreviations

IPBSS: Intermingled Phlegm and Blood Stasis Syndrome
CHD: Coronary heart disease
CM: Chinese medicine
AHP: Analytic hierarchy process
ACC: American College of Cardiology
AHA: American Heart Association
STEMI: ST-elevation myocardial infarction
NSTEMI: Non-ST-elevation myocardial infarction
PCI: Percutaneous coronary interventional
CABG: Coronary artery bypass grafting
CTA: Computed tomography angiography
ROC: Receiver operator characteristic
T2DM: Type 2 diabetes mellitus
COPD: Chronic obstructive pulmonary disease
AUC: Area under the curve.
